# The Presence of Colony-Stimulating Factor-1 and Its Receptor in Different Cells of the Testis; It Involved in the Development of Spermatogenesis In Vitro

**DOI:** 10.3390/ijms22052325

**Published:** 2021-02-26

**Authors:** Alaa Sawaied, Eden Arazi, Ahmad AbuElhija, Eitan Lunenfeld, Mahmoud Huleihel

**Affiliations:** 1 The Shraga Segal Department of Microbiology, Immunology, and Genetics, Faculty of Health Sciences, Ben-Gurion University of the Negev, Beer Sheva 8410501, Israel; alaasa@post.bgu.ac.il (A.S.); edenaraz@post.bgu.ac.il (E.A.); abuelhija@gmail.com (A.A.); 2 The Center of Advanced Research and Education in Reproduction (CARER), Faculty of Health Sciences, Ben-Gurion University of the Negev, Beer Sheva 8410501, Israel; lunenfld@bgu.ac.il; 3 Department of OB/GYN, Soroka Medical Center, Beer Sheva 8410501, Israel; 4 Faculty of Health Sciences, Ben-Gurion University of the Negev, Beer Sheva 8410501, Israel

**Keywords:** testis, spermatogenesis, in vitro culture of spermatogonial cells, testicular autocrine/paracrine factors, Colony stimulating factor-1 (CSF-1)

## Abstract

Spermatogenesis is a complex process, in which spermatogonial cells proliferate and differentiate in the seminiferous tubules of the testis to generate sperm. This process is under the regulation of endocrine and testicular paracrine/autocrine factors. In the present study, we demonstrated that colony stimulating factor-1 (CSF-1) is produced by mouse testicular macrophages, Leydig, Sertoli, peritubular cells and spermatogonial cells (such as CDH1-positively stained cells; a marker of spermatogonial cells). In addition, we demonstrated the presence of CSF-1 and its receptor (CSF-1R) in testicular macrophages, Leydig, Sertoli, peritubular cells and spermatogonial cells of human testis. We also show that the protein levels of CSF-1 were the highest in testis of 1-week-old mice and significantly decreased with age (2–12-week-old). However, the transcriptome levels of CSF-1 significantly increased in 2–3-week-old compared to 1-week-old, and thereafter significantly decreased with age. On the other hand, the transcriptome levels of CSF-1R was significantly higher in mouse testicular tissue of all examined ages (2–12-week-old) compared to 1-week-old. Our results demonstrate the involvement of CSF-1 in the induction the proliferation and differentiation of spermatogonial cells to meiotic and postmeiotic stages (BOULE- and ACROSIN-positive cells) under in vitro culture conditions, using methylcellulose culture system (MCS). Thus, it is possible to suggest that CSF-1 system, as a testicular paracrine/autocrine system, is involved in the development of different stages of spermatogenesis and may be used in the development of future therapeutic strategies for treatment of male infertility.

## 1. Introduction

Macrophage colony stimulating factor (MCSF), also called CSF-1 is produced by vascular endothelial cells, and in the testis by Leydig cells, peritubular cells and peritubular macrophages [1,2,3]. It affects the functionality and/or the development of a wide range of cells in different tissues in the body through a specific receptor (CSF-1R). This receptor was identified on different cell types, such as mononuclear cell lineage, mononuclear phagocyte precursors, blood monocytes, tissue macrophages (including testicular macrophages), dendritic cells, osteoclasts, trophoblasts, Langerhans cells, microglia, and spermatogonial cells [4,5,6,7,8,9,10,11,12,13,14]. Recently we showed that CSF-1R is also present on Leydig cells, Sertoli cells, and meiotic cells [14]. CSF-1 was shown to directly affect the proliferation of spermatogonial cells and Leydig cell steroidogenesis [13,15,16]. The lack of CSF-1 (*op/op* mice) led to sever depletion of testicular macrophages and a significant reduction in the levels of circulating and testicular testosterone [16], circulating luteinizing hormone (LH), follicular stimulating hormone (FSH), the number of viable spermatozoa and fertility [15,16,17]. Spermatogenesis is the process of spermatogonial stem cells (SSCs) proliferation and differentiation in the seminiferous tubules to generate sperm [18,19,20]. This process is under the regulation of gonadotropin hormones (LH and FSH) that produce by the pituitary in response to gonadotropin releasing hormone (GnRH) which is secreted from the hypothalamus, and testosterone that is secreted from Leydig cells in response to LH [18,19,20]. In addition, testicular cells produce various autocrine/paracrine factors that affect the proliferation and/or differentiation of SSCs [18,19,20,21,22,23].

In our laboratory we established 3-dimensional (3D) in vitro culture systems that enable the proliferation and differentiation of spermatogonial cells to meiotic and postmeiotic stages, and in some cases to the generation of sperm [18,19,24,25,26,27,28]. Recently, we showed that interleukin-34, which affects target cells through CSF-1R, is present in testicular cells and was able to induce proliferation and differentiation of spermatogonial cells in vitro to meiotic and postmeiotic stages [14].

The aim of the present study was to examine the presence of CSF-1 and its receptor (CSF-1R) in mouse and human testicular cells, and to evaluate its capacity to induce spermatogenesis in vitro.

## 2. Results

### 2.1. Effect of Mouse Age on the Protein Levels of CSF-1 and on the Transcriptome Levels of CSF-1 and CSF-1R in Testicular Homogenates

**Figure 1:**
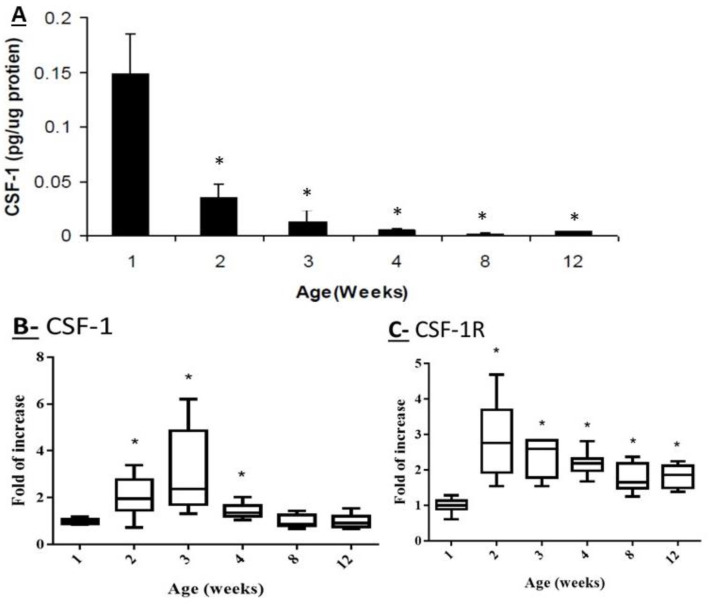
Effect of mouse age on the protein levels of CSF-1 and on the transcriptome levels of CSF-1 and CSF-1R in testicular homogenates. The protein levels of CSF-1 (**A**) were examined by specific ELISA and presented as pg CSF-1/μg total protein in the examined sample (pg/μg protein) ± SEM. The RNA transcriptome levels of CSF-1 (**B**) and CSF-1R (**C**) were examined by specific primers using qPCR analysis, and presented as fold of increase [median (minimum, maximum)] compared to β-actin of the same examined sample. These levels were examined in testicular homogenates of mice from different ages (1–12week-old). Number of mice used (n) for ELISA at each age was 10–12. For qPCR analysis n = 10 for each age. * *p* < 0.05.

Our results showed a high protein levels of CSF-1 in testicular homogenates of 1-week-old mice (Figure 1A). However, a significant and gradual reduction in protein levels of CSF-1 were examined in testicular homogenates of 2–12-week-old mice compared to 1-week-old mice, when the lowest levels examined at 12-week-old mice (Figure 1A). On the other hand, the higher transcriptome levels of CSF-1 were examined in testicular homogenates of 2–4-week-old mice compared to 1-week-old (Figure 1B), and thereafter (8–12-week-old), the transcriptome levels were decreased to become similar to 1-week-old mice (Figure 1B). The transcriptome levels of CSF-1R were significantly increased in testicular homogenates of 2–12-week-old mice compared to 1-week-old (Figure 1C).

### 2.2. Cellular Localization of CSF-1 in Mouse Testicular Cells

**Figure 2:**
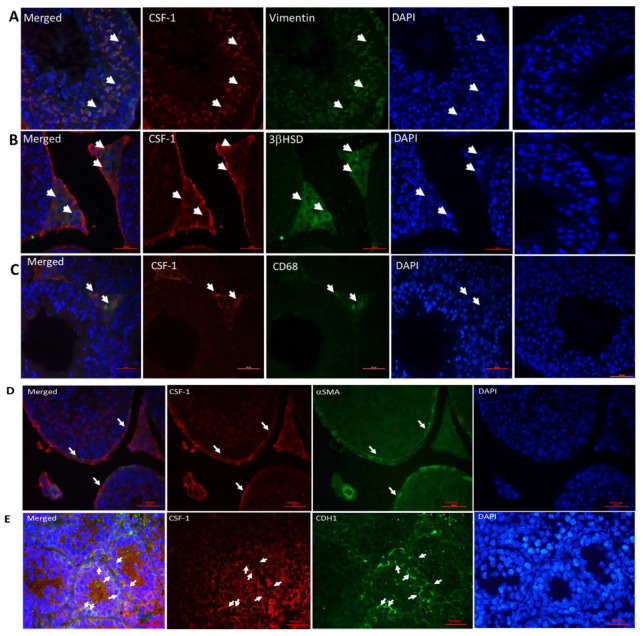
Cellular localization of CSF-1 in mouse testicular cells. Cellular localization of CSF-1 was examined in testis of adult mouse (6-week-old) for all markers except CDH1 which was examined in testes of 2-weeks-old mice, by double immunofluorescence staining using specific antibodies for CSF-1 (CSF-1; red color) and testicular cells such as Sertoli cells (**A**; vimentin—green color), Leydig cells (**B**; 3βHSD—green color), macrophages (**C**; CD68—green color), peritubular cells (**D**; αSMA—green color) and for spermatogonial cells (**E**; CDH1—green color). DAPI was also used to show the nucleus of the cells (DAPI; blue staining). Positive double immunofluorescence staining is presented in merged (orange color). Negative control (NC) staining of the tissues was performed as described in the Materials and Methods section and did not show staining of the examined markers. Arrows indicate the specific cells stained for each marker in the stained tissues (×400, scale bar: 100 µm).

Our results show the presence of CSF-1 in mouse testicular such as Sertoli cells (vimentin-positive cells, Figure 2A), Leydig cells (3βHSD-positive cells, Figure 2B), macrophages (CD68-positive cells, Figure 2C), peritubular cells (aSMA-positive cells, Figure 2D) and spermatogonial cells (CDH1-positive cells, Figure 2E).

### 2.3. Cellular Localization of CSF-1 and CSF-1R in Human Testicular Cells

**Figure 3:**
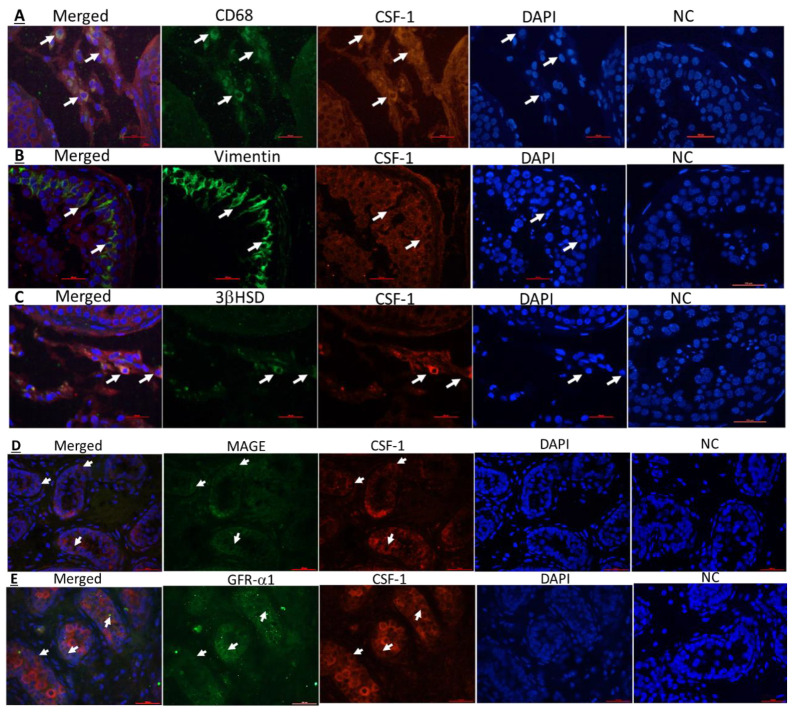

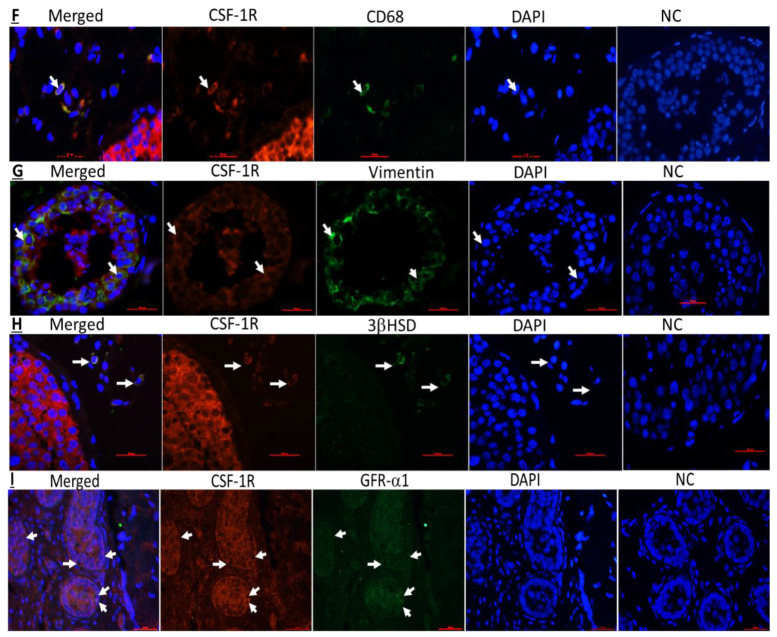
Cellular localization of CSF-1 and CSF-1R in human testicular cells. Cellular localization of CSF-1 (**A**–**C**) and CSF-1R (**F**–**H**) were examined in human testicular sections from patients with normal spermatogenesis and CSF-1 (**D**,**E**) and CSF-1R (**I**) were examined in human testicular sections of prepubertal boy. Double immunofluorescence staining was performed using specific antibodies for CSF-1 (CSF-1; red color) and testicular cells such as macrophages (**A**; CD68—green color), Sertoli cells (**B**; vimentin—green color), Leydig cells (**C**; 3βHSD—green color) and spermatogonial cells (**D**; MAGE-**A**,**E**; GFR-a1—green color), or for CSF-1R (CSF-1R; red color) and testicular macrophages (**F**; CD68—green color), Sertoli cells (**G**; vimentin—green color), Leydig cells (**H**; 3βHSD—green color) and spermatogonial cells (**I**; GFR-a1—green color). Positive double immunofluorescence staining is presented in merged (orange color). DAPI was also used to show the nucleus of the cells (DAPI; blue staining). Negative control (NC) staining of the tissues was performed as described in the Materials and Methods section and did not show staining of the examined markers. Arrows indicate the specific cells stained for each marker in the stained tissues (×400, scale bar: 100 µm).

Our results, using double immunofluorescence staining for CSF-1 or CSF-1R and the markers for the examined cells, show that CSF-1 and CSF-1R are present in human testicular macrophages (Figure 3A,F; respectively), Sertoli cells (Figure 3B,G; respectively) and Leydig cells (Figure 3C,H; respectively). In addition, we show that human spermatogonial cells (MAGE-A- and GFR-a1-positive stained cells; markers for spermatogonial cells) express CSF-1 (Figure 3D,E) and GFR-a1 cells express CSF-1R (Figure 3I).

### 2.4. Development of Colonies and Different Stages of Spermatogenesis from Spermatogonial Cells In Vitro

**Figure 4:**
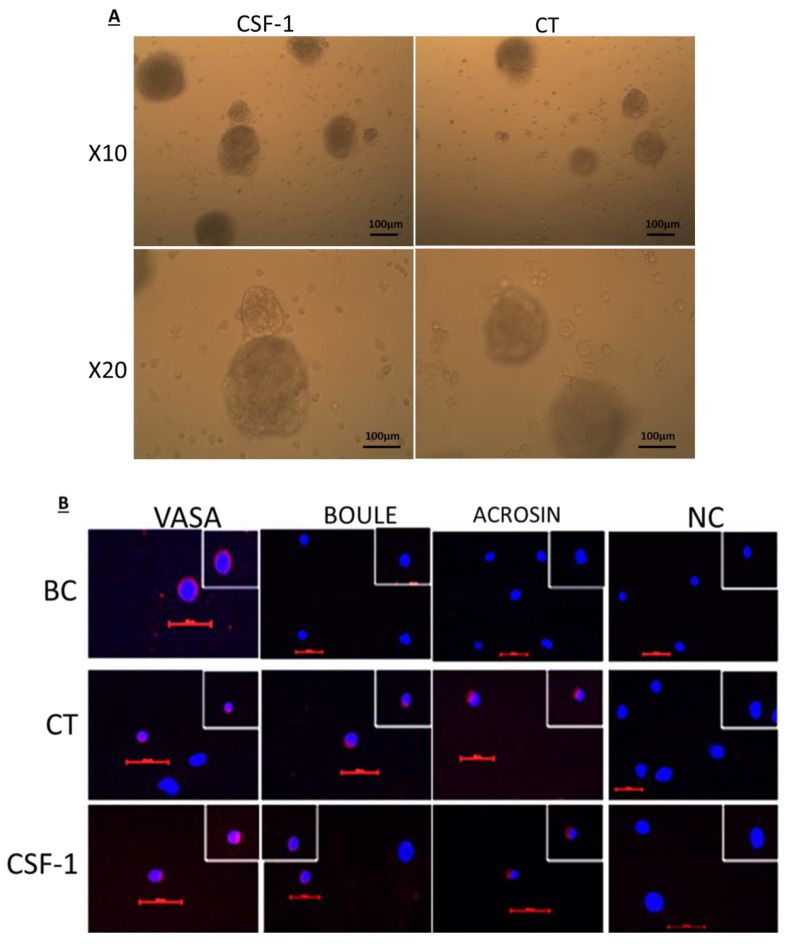

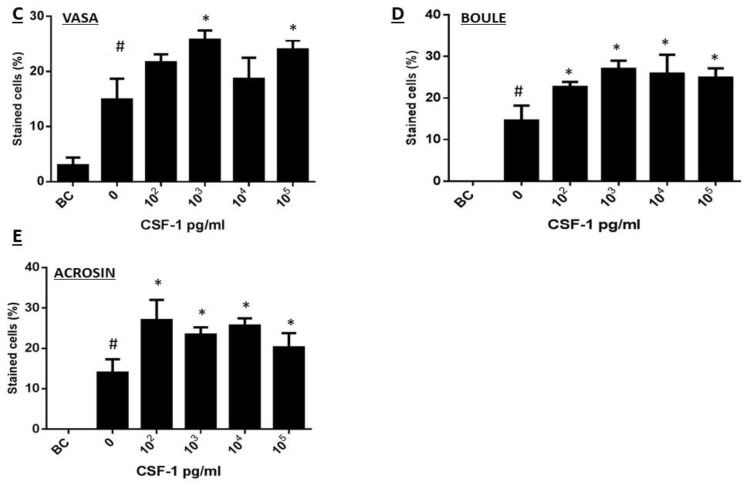
Effect of CSF-1 on the development of colonies and different stages of spermatogenesis from spermatogonial cells in vitro. After 4-weeks of culture, we identified the development of colonies/clusters of cells/organoids at different morphologies, which were not affected by the absence (control; CT) or presence of CSF-1 (CSF-1) (**A**). Colonies are presented in two magnifications (×10 and ×20) (**A**). The developed cultures were collected and examined for the presence of premeiotic cells (VASA), meiotic cells (BOULE) or postmeiotic cells (ACROSIN) by immunofluorescence staining using antibodies specific for each cell marker (**B**). Negative control (NC) staining of the cells was performed as described in the Materials and Methods section and did not show staining of the examined markers. The effect of different concentrations of CSF-1 in the culture (0–100,000 pg/mL) on the development of VASA, BOULE and ACROSIN was calculated and presented as percentages of the stained cells for VASA (**C**), BOULE (**D**) and ACROSIN (**E**). Magnification ×400 and scale bar: 100 µm. # *p* < 0.05 compared to before culture (BC), * *p* < 0.05 compared to absence of CSF-1 (0 pg/mL).

Culture of cells isolated from seminiferous tubule of 7-day-old mice in vitro in methylcellulose in the absence (control; CT) or presence of CSF-1 (CSF-1) lead to development of clusters/colonies of cells (Figure 4A). Cells isolated from the cultures after 4 weeks in the absence (control; CT) or presence of CSF-1 (CSF-1), stained positively for VASA (a marker for spermatogonia), BOULE (a marker for spermatocytes) and ACROSIN (a marker for spermatid- meiotic/postmeiotic stages) compared to cells before culture (BC) that stained only for VASA (Figure 4B). Using inverted microscope, we could not identify histological/size differences in the cells and colonies developed in the CT and CSF-1-treated cultures (CSF-1).

### 2.5. CSF-1 Induced the Development of Spermatogonial Cells to Different Stages of Spermatogenesis In Vitro

Our results show that cells before culture (BC) stained only for VASA (Figure 4C), but not for BOULE (Figure 4D) or ACROSIN (Figure 4E). However, after 4 weeks of culture in MCS, a significant increase the percentage of cells that positively stained for VASA (Figure 4C), BOULE (Figure 4D) and ACROSIN (Figure 4E) were detected compared to BC. On the other hand, addition of CSF-1 (100–10000 pg/mL) for 4 weeks to the MCS significantly increased the percentage of cells that positively stained for VASA (optimal effect was measured at 1000 pg/mL)

(Figure 4C), BOULE (optimal effect was measured at 100 pg/mL) (Figure 4D), and ACROSIN (optimal effect was measured at 1000 pg/mL) (Figure 4E) compared to control (0 pg/mL).

## 3. Discussion

Our results show for the first time that CSF-1 protein levels in mouse testis decrease with age. However, the transcriptome levels of CSF-1 were significantly increased in testis of 2–4-week-old mice compared to 1-week-old, and thereafter were decreased to similar levels of 1-week-old. On the other hand, the transcriptome levels of testicular CSF-1R were significantly increased with mouse age. These results may suggest a biological role of CSF-1 system (CSF-1 and CSF-1R) in the development of the testis and in testicular functions including spermatogenesis. This suggestion is supported by studies used mice lacked CSF-1 or CSF-1R [14,15,29,30]. Indeed, the absence of CSF-1 led to a significant reduction in the levels of circulating and intratesticular testosterone, circulating LH, FSH, in the count of viable spermatozoa and in mating capacity [15,16,31]. Furthermore, testicular macrophages were shown to severely deplete through development in mouse lacked CSF-1 [1,15,16,31]. Also, the cell-cell interaction and the association between testicular macrophages and Leydig cells were affected and led to a significant reduction in Leydig cell steroidogenesis [15,16,17,31,32]. The lack of CSF-1R in mice, showed a similar phenotype to CSF-1 lack [15,29,33]. In addition, changes in the levels of CSF-1 system with age, may suggest possible involvement of endocrine factors, such as gonadotropins and testosterone, in the regulation of testicular CSF-1 system under physiological conditions. Indeed, a correlation between CSF-1 levels and GnRH and gonadotropins was demonstrated. A reduction in the levels of LH and FSH were examined in mouse lacked CSF-1 [15,16,31]. It was suggested that CSF-1 could act through the hypothalamus and pituitary to control the production of GnRH and thus to affect the production of LH and FSH [15,16,31]. This effect was related to sever depletion of testicular macrophages which affect the Leydig cell steroidogenesis.

The differences in the protein levels of CSF-1 and its transcriptome levels with age could be related to their distinct regulation at the levels of transcription and translation and/or their stability at the different ages. In addition, the transcriptome levels of CSF-1 and CSF-1R behaved similarly, even though the transcriptome levels of CSF-1R remain higher at 8-12-week-old mice compared to 1-week-old, while at those ages the transcriptome levels of CSF-1 were similar to 1-week-old mice. This may indicate that the higher levels of CSF-1R enable more CSF-1 functionality at its lower levels at the adult ages.

To the best of our knowledge, we demonstrate for the first time, the presence of CSF-1 in mouse testicular Sertoli cells, interstitial macrophages and spermatogonial cells (CDH1), and confirmed the presence of CSF-1 in mouse peritubular cells and Leydig cells as previously published [2,3]. Furthermore, we showed for the first time the presence of CSF-1R in mouse Sertoli cells, Leydig cells and meiotic cells (BOULE), and confirmed the presence of CSF-1R in spermatogonial cells (positively stained for the marker CDH1), as previously demonstrated [13,14]. We also demonstrate the presence of CSF-1 and CSF-1R in human testicular cells; macrophages, Leydig cells, Sertoli cells and spermatogonial cells. Thus, our results may suggest a crucial role of CSF-1 in the cross talk between Leydig cells and testicular macrophages to regulate normal steroidogenesis by Leydig cells, and also in regulating the development of spermatogonial cells. This suggestion is supported by studies used mice lacked CSF-1 or CSF-1R that lead to sever depletion of macrophages and reduction in steroidogenesis of Leydig cells [15,16,31,32]. The presence of CSF-1 and CSF-1R in tubular cells; mainly Sertoli and spermatogonial cells may suggest the involvement of CSF-1 in the cell-cell interaction between to provide optimal conditions to normal development of spermatogenesis. All these results may suggest CSF-1 as a crucial factor involved in the normal niche of spermatogonial cells as well as in regulation the secretion of gonadotropins and testosterone for developing normal spermatogenesis.

The possible role of CSF-1 in the development of normal spermatogenesis was examined in vitro culture of spermatogonial cells in methylcellulose culture system (MCS). Our results showed that in vitro culture of isolated seminiferous tubules in MCS for 4 weeks in the presence of different concentrations of CSF-1 significantly induced the development of VASA cells compared to control (absence of CSF-1), and induction the development of meiotic and postmeiotic cells. These results confirm the possible role of CSF-1 in development of different stages of spermatogenesis. The effect of CSF-1 in vitro could be directly on the premeiotic cells (spermatogonia) and meiotic cells (BOULE) which express CSF-1R [13,14] and or indirectly through induction the somatic cells present in the culture (Sertoli, peritubular and Leydig cells) to produce various factors and provide optimal niches that involved in the development of spermatogonial cells to different stages of spermatogenesis.

In summary, we demonstrated the presence of CSF-1 and CSF-1R in testicular somatic cells and spermatogonial cells, and its capacity to induce spermatogonial cells to different stages of spermatogenesis *in vitro*. These results suggest CSF-1 system as a crucial system in the process of spermatogenesis. The limitation of this in vitro system is the inability to generate mature sperm, even though in some studies we were able to induce the development of sperm-like cells but this was not consistent and the yield was very low. In addition, the specific factors/conditions and the sequence of their addition into the culture is still unknown. Optimization this system may open future therapeutic strategies for treatment of male infertility.

## 4. Materials and Methods

### 4.1. Animals and Human Materials

The present study was confirmed by the Ben-Gurion University Ethics Committee for Animal Use in Research (No. IL-16-04-2018). Hsd:ICR (CD-1^®^) (ICR; Institute of Cancer Research) mice at different ages (1–12-week-old) were purchased from Harlan Laboratories (Jerusalem, Israel). Human testis materials: Local Institutional Ethics Committees approved the study (#5011 and # 4538).

### 4.2. Chemicals and Reagents

Roswell Park Memorial Institute Medium (RPMI)-1640 media, penicillin, streptomycin, and fetal calf serum (FCS) were purchased from Beit HaEmek Biological Industries (Beit HaEmek, Israel). Collagenase IV (from clostridium histolyticum) and DNAase were obtained from Sigma (St. Louis, MI, USA). The levels of CSF-1 protein were determined by enzyme-linked immunoassay (ELISA) (DuoSet^®^, DY-416-05, R&D Systems, Minneapolis, MN, USA).

### 4.3. Testicular Homogenates

Testicular tissues from different ages (1–12-week-old mice) were homogenized for protein examination and RNA extraction according to Huleihel et al., 2013 [34].

### 4.4. Extraction of Total RNA for Real-Time PCR Analysis

Extraction of total RNA from testicular tissue of different ages of mice was performed by using a Dynabeads RNA direct kit (Dynal Biotech, Oslo Norway) as previously described [34].

Real-time quantitative PCR amplification of total cDNA (500 ng/sample) used specific primers of the different sequences: CSF-1-Fw5′-CCCATATTGCGACACCGAA, Rw 5′-AAGCAGTAACTGAGCAACGGG. CSF-1R: –FW 5′-GCATACAGCATTACAACTGGACCTACC, Rw 5′-CAGGACATCAGAGCCATTCACAG, and β-ACTIN: Fw: 5′ GCATTGTTACCAACTGGGAC, Rw: 5′-GGTCTCAAACATGATGTGGG. The reactions were performed according to our previous study using the protocol for the Absolute qPCR SYBR Green mix (ABgene House, Blenheim Road, Epsom, UK) as described previously [27]. The relative quantity of the gene transcriptome was analyzed using the 2^−∆∆Ct^ method [35]. The results were expressed as fold of increase related to the β-ACTIN of the same examined sample.

### 4.5. Immunofluorescence Staining of Testicular Tissue

Testicular tissues from mice and human testicular tissue with normal spermatogenesis were fixated in Bouins solution [form mouse tissue and prepubertal boy (12-year-old)] or formalin (for adult human), and paraffin-embedded. Testicular tissues were cut into 4 μm sections and stained according to Arafat., M, 2017 [35]. For immunofluorescence staining of CSF-1 (MCSF), polyclonal rabbit anti-mouse MCSF antibodies (ab99178, abcam, Cambridge, UK) were used as primary antibodies and for CSF-1R we used polyclonal rabbit anti-mouse CSF-1R antibody (LS-C164350, LifeSpan BioSciences, Seattle, WA, USA) (1:200 final dilution). For Sertoli cell marker-vimentin, we used mouse monoclonal anti mouse/rat/human (Sc-373717, Santa Cruz Biotechnology, Inc., Santa Cruz, CA, USA; 1:200 final dilution). For Leydig cell marker—3β-hydroxysteroid dehydrogenase (3βHSD); we used polyclonal goat anti-mouse/human/rat 3βHSD (Sc-30820, Santa Cruz Biotechnology, 1:100 final dilution), for peritubular cell marker -alpha smooth muscle actin (αSMA), we used polyclonal goat anti-mouse/human (αSMA), (Ab21027, Abcam, Cambridge, UK). For macrophage cells marker—CD68, we used monoclonal mouse anti-mouse/rat/human (sc-20060, Santa Cruz Biotechnology, 1:100 final dilution). For the spermatogonial cell marker –CDH1, we used polyclonal goat anti-mouse/human Cadherin (AF748, R&D Systems, Minneapolis, MN, USA), For GFR-a1, we used goat polyclonal GFRα-1 antibody (Santa Cruz, sc-6157), and for MAGE-A, we used mouse monoclonal MAGE-A antibody (sc-20034). Secondary antibodies; we used fluorescein-conjugated antibodies (Cy3, donkey anti-rabbit (1:1000 dilution; Jackson ImmunoResearch, West Grove, PA, USA) and Dylight 488, rabbit anti-goat antibodies (1:100 dilution; KPL, Milford, MA, USA), Dylight 488, rabbit anti-mouse antibodies (1:100 dilution; KPL, Milford, MA, USA). Secondary antibodies were incubated at room temperature for 1 h. Thereafter, slides were washes with PBS and were dried and DAPI was added (Santa Cruz Biotechnology).

### 4.6. Isolation of Testicular Tubular/Spermatogonial Cells

The tubular cells were enzymatically isolated from the testes of the 7-day-old ICR mice. The testicular cell suspensions were obtained as described by Elhija et al., 2012 [24]. The suspension cells were filtered through a sterile cell strainer (70 µM; BD Biosciences) and washed with RPMI (Roswell Park Memorial Institute Medium-1640 (RPMI) (Beit HaEmek Biological Industries (Beit HaEmek, Israel) and centrifugation at 100× *g* for 5 min. After centrifugation, the media were removed and the pellet of the cells in the bottom of the tube was suspended in 1 mL of fresh RPMI, and were counted under phase-contrast microscopy in a Neubauer counting chamber.

### 4.7. Effect of CSF-1 on the Development of Isolated Spermatogonial Cells In Vitro in Methylcellulose Culture System (MCS)

The isolated tubular cells were cultured (2 × 10^5^ cells/well/500 µL) in 3D-methylcellulose culture system according to Sawaied, et al., 2020 [14]. Briefly, the cells were cultured in methylcellulose (R&D systems, Minneapolis, MN, USA) (42%), RPMI (32%)-containing FCS (fetal calf serum) (25%) in the absence [0 (control, CT)] or presence of CSF-1 (10–100,000 pg/mL) and incubated in CO_2_ incubator in 37 °C for 4 weeks. After 10–14 days of the culture, fresh media with or without the relevant concentration of CSF-1 were added in 50 μL/well. At the end of the incubation (after 4 weeks) the developed cells were collected as previously described [14]. Briefly, 0.5 mL of PBS was added to the methylcellulose in each well, pipetting, and the suspension was collected. After centrifugation (1600 rpm for 10 min), the cells at the bottom of the tubules were collected, and smeared on a slide for histological examination and/or collected for RNA extraction.

### 4.8. Evaluating the Spermatogenic Types Developed in MCS by Immunofluorescence Staining

The fixed (cold methanol) cells were examined for the presence of different spermatogenic cells by immunofluorescence staining. For evaluating the premeiotic cells (VASA), meiotic cells (BOULE) and postmeiotic cells (ACROSIN), immunostaining was performed as previously described [14]. For VASA we used as primary antibody polyclonal rabbit anti-mouse (NBP224558, Novus Biologicals, Centennial, CO, USA;1:50 final dilution), for BOULE we used mouse monoclonal antibody (sc-166660, Santa Cruz Biotechnology, Inc.; 1:100 final dilution) and for ACROSIN we used polyclonal rabbit anti-mouse (NBP2-14260, Novus Biologicals, 1:2000 final dilution). Before the primary antibodies were applied, the non-specific background was blocked with PBS containing 4% FCS/BSA. The relative secondary antibodies were used: Fluorescein-conjugated antibodies (Cy3; donkey anti-rabbit, 1:1000 dilution; Jackson ImmunoResearch) and Dylight 488, rabbit anti-mouse antibodies, 1:100 dilution (KPL, Milford, MA, USA), were used for visualization of the signal according to the suppliers’ directions. After 1 h of incubation, the slides were washed in PBS and subsequently subjected to DAPI staining (Santa Cruz Biotechnology, Inc. Santa Cruz, CA, USA). Negative controls were included for each specimen using PBS containing FCS/BSA/relevant IgG isotype instead of the primary antibodies. Slides were examined for staining using the Nikon eclipse 50i microscope

### 4.9. Data Handling and Statistical Evaluation

Each culture condition (in a single experiment) was tested in at least three wells, and each experiment was repeated at least three times. The plotted data are the means calculated from a minimum of three independent experiments. The standard error (SEM) represents the variability between independent experiments. The Student’s t-test was used for the estimation of statistical significance and *p* values below 0.05 were considered significant. For gene expression data, we used Manny-Whitney test for statistical analysis, and presented the data as median (minimum, maximum).

## Data Availability

The data that support the findings of this study are available from the corresponding author upon reasonable request.
